# Moss-Derived Mesoporous Carbon as Bi-Functional Electrode Materials for Lithium–Sulfur Batteries and Supercapacitors

**DOI:** 10.3390/nano9010084

**Published:** 2019-01-10

**Authors:** Wen Lei, Haipeng Liu, Junlei Xiao, Yang Wang, Liangxu Lin

**Affiliations:** 1 The State Key Laboratory of Refractories and Metallurgy, and Institute of Advanced Materials and Nanotechnology, Wuhan University of Science and Technology, Wuhan 430081, China; lhpWUST@gmail.com (H.L.); pvt516987834@gmail.com (J.X.); 2 Hefei Guoxuan High-Tech Power Energy Co., Ltd., 599 Daihe Road, Hefei 230000, China; 1152036@dhu.edu.cn; 3 ARC Centre of Excellence for Electromaterials Science, Intelligent Polymer Research Institute, Institute of Innovative Materials (AIIM), Innovation Campus, University of Wollongong, Wollongong 2500, Australia

**Keywords:** moss, biomass derived carbon, lithium–sulfur batteries, supercapacitors

## Abstract

In this work, we reported a moss-derived biomass porous carbon (MPC) as a bi-functional electrode material for both the lithium–sulfur battery and the supercapacitor. The MPC was prepared from a high-temperature calcination procedure using the moss as the carbonaceous precursor. Using NaOH, the MPC was activated to give a mesoporous structure with a high specific surface area (1057.1 m^2^ g^−1^) and large pore volume (0.72 cm^3^ g^−1^). When it was used as the cathode material in lithium–sulfur batteries, the MPC material realized a sulfur loading and exhibited a remarkably improved electrochemical performance, i.e., a high discharge capacity of 1070 mAh g^−1^ at 0.1 C. This activated MPC also worked well as a capacitive electrode in supercapacitors, demonstrating a high specific capacitance of 332 F g^−1^ (scan rate of 1.0 A g^−1^) and a high capacity retention > 97% in a long-term cycle of 1000 charge/discharges. This work demonstrated a facile method for the utilization of activated waste biomass material for future clean energy applications.

## 1. Introduction

To satisfy the increasing demand for cheap and efficient clean energy technologies, the development of low cost rechargeable energy storage devices with high specific energy and high stability is highly sought after [1,2]. Among various secondary batteries developed so far, the lithium–sulfur (Li-S) batteries assembled by lithium metal anodes and sulfur cathodes are highly competitive and are promising energy storage devices [3]. Theoretically, they have very high specific capacity (1675 mAh g^−1^) and specific energy (2600 Wh Kg^−1^), and an average operating voltage of 2.1 V, which is superior to the current commercial lithium–ion batteries [4]. The manufacture of such Li-S batteries is also affordable because of the low-cost and the abundant resource of the raw material [5].

Despite these advantages, the practical application of Li-S batteries is still limited by several technical weaknesses, such as the high solubility of the lithium polysulfide and the formation of unfavorable intermediate products in electrolytes during the discharge processes. The latter reduces the utilization of raw materials, increasing the cost and causing irreversible loss of capacity [5,6,7,8]. To overcome these challenges, several strategies have been explored, including the use of novel cathode materials [9,10,11,12,13], the surface protection of the lithium anode [14,15,16,17], the modification of the electrolyte [18,19,20], and the optimization of the cell configuration [21,22,23]. In these approaches, the development of a suitable conductive porous carbon material is highly promising and can be considered as one of the most direct ways to improve the performance of the Li-S battery [24]. For such purposes, commercial carbon black [25], carbon nanotubes, and other carbon-based materials [26] have been extensively tested as cathode materials for Li-S batteries. However, for most of these carbon materials, their fabrication processes should be further simplified to reduce the cost of scale-up production.

Unlike the above traditional materials, the emerging biomass derived carbon materials might be an ideal carbon electrode for high-performing Li-S batteries, owing to their low production costs, renewable/environmentally friendly resources, and adjustable physical/chemical properties [27,28]. The activated bio-mass materials could be readily used in Li-S batteries [29,30]. For example, Chung et al. employed a natural material obtained from domestic waste as the cathode carrier to achieve a high discharge capacity of 1327 mAh g^−1^ (scan rate of 0.05 mV s^−1^) and a good stability (97% Columbic efficiency retention after 100 cycles) [31]. Ji et al. prepared mesoporous sulfur/carbon composites using a melting process [9]. The large pore volume and the uniform mesoporous carbon structure allowed the successful encapsulation of sulfur into the pore space, yielding a high capacity of 830 mAh g^−1^ (at 0.1 C), with acceptable stability (71% capacity remained after 100 cycles). These achievements encouraged us to develop the alternative biomass-derived carbon material, the moss, as the electrode material for the Li-S battery. As we know, moss is an aquatic plant and a worldwide water contaminant. Utilization of the cheap and abundant moss in valuable Li-S batteries and similar energy storage approaches has positive significance in promoting clean energy technologies. In the long-term perspective of environmental conservation and sustainable development, searching for valuable biomass derived carbon materials and exploring their applications in other fields, such as supercapacitors, has also been regarded as an alternative choice. The natural superiorities of biomass derived carbon materials, e.g., well-developed porous structure [32], large specific surface area [33], and different types of heteroatoms doping [34], endow them with unparalleled advantages when used as electrode materials for high performance supercapacitors [35].

In this paper, we demonstrated a high-performing (e.g., high discharge specific capacity, good cycling stability and rate performance) Li-S battery using the cathode material from the moss-derived porous carbon (MPC) biomass. This MPC with a high surface area was activated with a conventional approach (heating reaction of the carbon contained materials with NaOH), which is also capable of providing a high specific capacitance when used in supercapacitors. Given the natural porous structure and abundant resources, this work demonstrated that moss-derived porous carbons can be a promising electrode material for energy storage and conversions.

## 2. Experimental

### 2.1. Preparation of MPC and Activation Process

The MPC was obtained through the simple cleaning and high-temperature calcination of moss (Jin River, Tianjin, China). First, moss was ultrasonicated in deionized water and alcohol. It was then placed in a dry box and kept at 60 °C for 12 h. The dried moss was calcined at 800 °C (heating rate: 5 °C min^−1^) for 3 h in Ar atmosphere, and then naturally cooled down. The as-obtained product was referred to as MPC. Next, NaOH was employed as the activator to increase the specific surface area and pore volume of the MPC. Briefly, MPC was dispersed into 1.0 M NaOH solution (NaOH/MPC mass ratio of 3:1) and stirred for 12 h. The mixture was filtered and then calcined at 800 °C in Ar for another 2 h. After that, the product was rinsed with 1.0 M hydrochloric acid (HCl) and deionized water until the pH was 7.

### 2.2. Characterization

Morphologies of the MPC sample were characterized using scanning electron microscopy (SEM) and transmission electron microscopy (TEM). The structure of the MPC sample was investigated using Raman spectroscopy and X-ray diffraction (XRD). Raman spectra were performed on a LabRam HR800 spectrometer (HORIBA, Kyoto, Japan) with a 532 nm laser excitation. XRD was employed to confirm the graphite crystal structure using Cu Ka radiations (λ = 0.15406 nm) at a speed of 2° min^−1^. The X-ray photoelectron spectroscopy (XPS) was performed on a Kratos AXIS Ultra DLD X-ray photoelectron spectroscopy (Kratos, Manchester, UK) (excitation source of Al Ka). The binding energy of XPS was calibrated based on C1s (284.5 eV). The specific surface area and pore size distribution was analyzed from the N_2_ adsorption–desorption isotherms with ASAP 2020 (Micromeritics, Norcross, GA, USA). The sulfur content was determined using thermogravimetric analysis (TGA) under N_2_ atmosphere.

### 2.3. Electrode Preparation and Electrochemical Measurements

Preparation of the S@MPC: The as-prepared MPC sample was mixed with sulfur at the weight ratio of MPC/sulfur = 1:3. After sufficient grounding in a mortar, the mixture was placed into the reaction vessel protected with Ar, and then heat treated at 155 °C for 12 h to give S@MPC. The cathode for the Li-S battery test was fabricated through a conventional slurry-coating process. Briefly, the S@MPC, the polyvinylidene fluoride (PVDF) binder, and the acetylene black were mixed and ground at the mass ratio of 8:1:1. After adding appropriate amounts of N-Methyl-2-pyrrolidone (NMP), the slurry was uniformly spread onto an aluminum foil and dried. The resulting pole piece was then fashioned into a round piece with diameter of 1.2 cm and used as sulfur electrodes. The anode was made of the lithium metal. The electrolyte was 1.0 M lithium bis (trifluoromethanesulfonyl) imide (LiTFSI) in 1,3-dioxolane (DOL) and 1,2-dimethoxyethane (DME) (1:1 by volume). The 2025-type coin cell was assembled inside an Ar-filled glove box, and the Galvanostatic charge/discharge measurement was performed on a multichannel battery tester (BTS-5V5mA, Neware, Shenzhen, China). All the electrochemical tests were performed at room temperature. Electrodes for the supercapacitor test were prepared by mixing MPC powders with the super P and the polytetrafluoroethylene (PTFE) emulsion at a weight ratio of 8:1:1. Then, 6.0 M KOH solution was used as the electrolyte in a three-electrode test system. The mass load of each electrode was approximately 3.0 mg.

## 3. Results and Discussion

**Figure 1:**
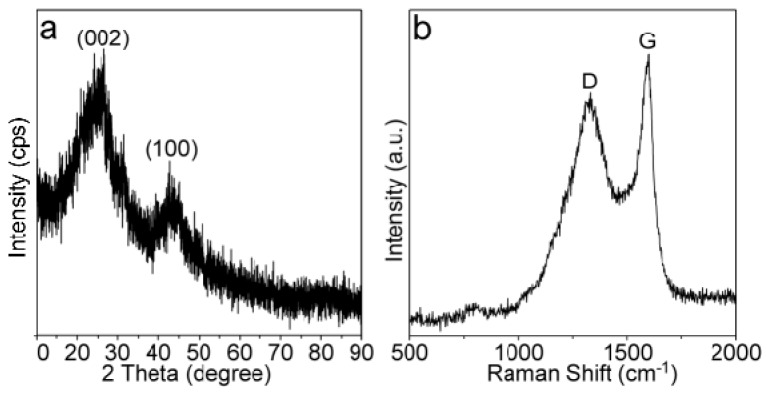
(**a**) X-ray diffraction (XRD) pattern and (**b**) Raman spectrum of the as-prepared moss-derived biomass porous carbon (MPC).

The XRD pattern of the as-prepared MPC is shown in Figure 1a, giving two broad diffraction peaks at around 26.2 and 42.8°. These two peaks can be ascribed to the (002) and (100) diffractions of the graphite structure (ICDD cards: 41-1487). The weak and broad diffraction peaks meant the long-range disorder of the graphite structure due to the random packing of the sheets (would be further clarified). Nevertheless, our Raman analyses suggested that the local domain of the MPC was highly graphitized with a continuous sp^2^ benzene-ring structure. As suggested in Figure 1b, the D and G bands of the MPC were identified at around 1321 and 1597 cm^−1^, corresponding to the breathing mode of defect (disordered) carbon atoms and the vibration of lattice carbon atoms, respectively. The I_D_/I_G_ (relative intensity ratio of the D and G bands) could reflect the extent of graphitization and alignment of the graphene planes in the carbon-based materials [13]. The I_D_/I_G_ value in Figure 1b is larger than 1 considering the contribution of the G peak by a D2 peak at around 1620 cm^−1^, indicating a high l structure disorder of the MPC material. Compared with highly reduced graphene and graphite materials (e.g., 1587 cm^−1^) [35], the G band of our sample was slightly blue shifted probably owing to the chemical doping, i.e., the out-of-plane doping, which increased the dangling strength of the C–C/C=C (will be discussed later).

**Figure 2:**
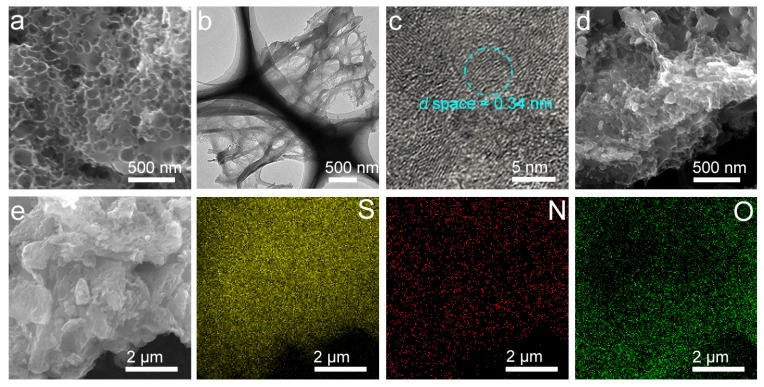
(**a**–**c**) Scanning electron microscopy SEM (**a**) and bright field transmission electron microscopy (TEM) images (**b**,**c**) of the MPC; (**d**) SEM image of the S@MPC; (**e**) the SEM and corresponding S, N, and C mappings of the S@MPC.

Figure 2a–c show the SEM and TEM images of the MPC. According to Figure 2a, the MPC has the evident porous surface structure, which is further confirmed by the TEM observation (Figure 2b). Our TEM observations also confirmed the (002) lattice of around 0.34 nm from many graphite domains with some dislocated interlayers (Figure 2c). Such a porous structure explains well the broad and weak XRD diffraction peaks (Figure 1a) and the strong D mode of the MPC (Figure 1b). Existence of this porous structure, in principle, can improve the electrochemical performance of the lithium–sulfur battery by increasing the specific surface area and pore volume of the electrode material. Unlike the MPC material, pores of the S@MPC were not clear (Figure 2d), suggesting that our preparation had efficiently filled the sulfur particles into the pore space of the MPC material. To further verify the distribution of sulfur, oxide, and carbon in the S@MPC sample, we carried out the energy dispersive spectroscopic (EDS) elemental mapping analysis (Figure 2e), confirming the homogenous dispersion of these elements in the S@MPC sample. The O and N were derivated from the raw material of moss and incorporated into the sp^2^ C–C/C=C structure.

**Figure 3:**
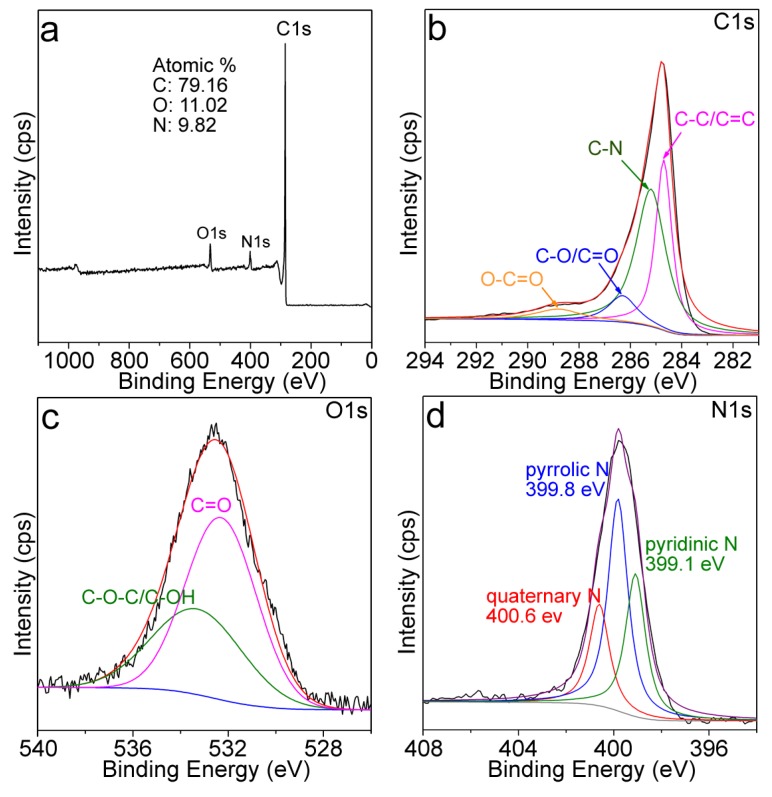
(**a**) X-ray photoelectron spectroscopy (XPS) Full survey; (**b**) C1s, (**c**) O1s, (**d**) N1s of the MPC.

Figure 3a shows the XPS full survey of the MPC, giving the atomic ratios of O and N at around 11.02% and 9.82%, respectively. C1s XPS spectrum of the MPC (Figure 3b) was composed of four binding energy peaks corresponding to the C–C/C=C (284.7 eV), C–N (285.3 eV), C–O or C=O (286.4 eV), and O–C=O (288.9 eV), respectively [31]. The existence of the O-containing groups was also confirmed by the O1s XPS spectrum (Figure 3c). C=O and the N–C structure in the MPC was complex, but could be recognized as the pyridinic, pyrrolic, and quaternary N with binding energies at around 399.1, 399.8, and 400.6 eV, respectively (Figure 3d) [30].

**Figure 4:**
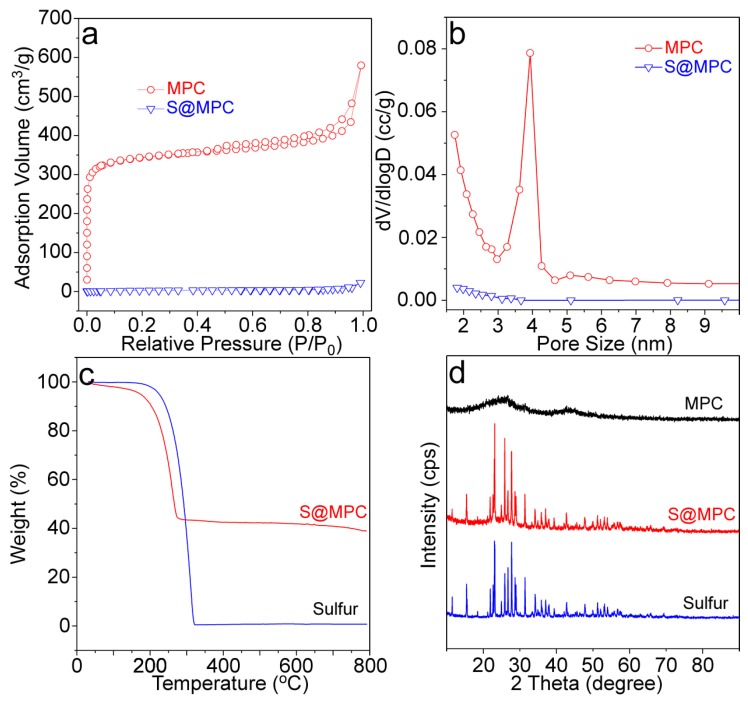
(**a**) N_2_ adsorption-desorption isotherms; (**b**) BJH pore size distribution; (**c**) TGA curves of the sulfur and S@MPC materials; (**d**) XRD patterns of the MPC, sulfur, and S@MPC materials. Note: XRD backgrounds of these samples were shifted for better comparison.

Figure 4a,b show the N_2_ adsorption–desorption isotherms and the pore size distributions of both the MPC and S@MPC. The adsorption–desorption isotherm of the activated MPC was a type IV isotherm, suggesting the existence of abundant mesopores (a pore volume of 0.72 cm^3^ g^−1^). The surface area calculated from the adsorption curve with the BET (Brunauer-Emmett-Teller) method was 1057.1 m^2^ g^−1^. The pore size distribution of the MPC derived from the desorption curve with the BJH (Barrett-Joyner-Halenda) method gave a pore size of around 3.5–4.0 nm. In contrast, the BET surface area of the S@MPC material was really low (10.5 m^2^ g^−1^), corresponding to the disappearance of the mesopore (Figure 4b). This phenomenon was reasonable, since both the large pores (see SEM images, Figure 2) and the mesopores were filled by the sulfur during the preparation.

The exact sulfur loading in the porous MPC sample was determined by the TGA technique (Figure 4c). At 200–320 °C, the TGA curves of both the sulfur and S@MPC showed obvious weight loss, corresponding to large decomposition of sulfur. According to this weight loss stage, the sulfur content in the S@MPC was determined to be 58 wt %. Figure 4d shows the XRD patterns of the sulfur and S@MPC. The XRD pattern of the MPC is also shown in this figure for better comparison. Diffractions from the crystallized sulfur (see the diffraction peaks from the sulfur raw material) were commonly found in the S@MPC, which were narrow and intense. In the XRD pattern of the MPC, diffraction peaks from initial MPC was nearly invisible owing to the strong diffractions from the crystallized sulfur.

**Figure 5:**
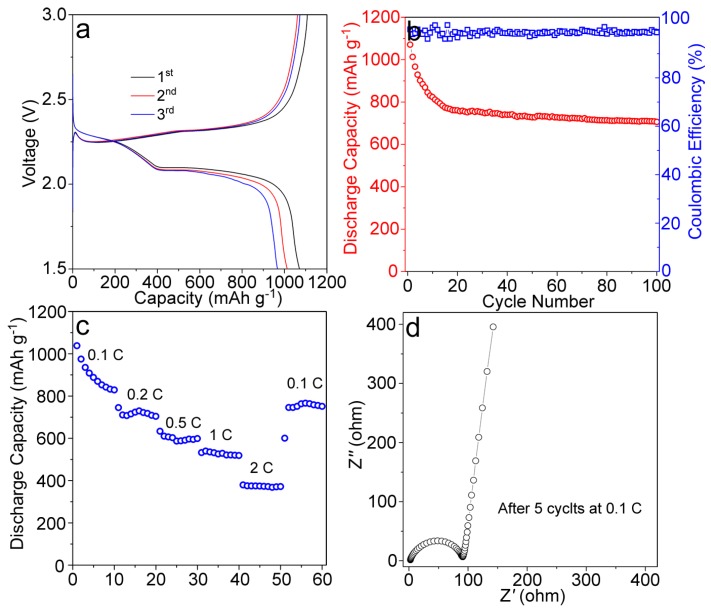
(**a**) Charge and discharge performance of the S@MPC electrode at 0.1 C; (**b**) Cycling performance and coulombic efficiency of the S@MPC electrode at 0.1 C; (**c**) Rate performance of the S@MPC electrode; (**d**) The Nyquist plots of S@MPC electrode.

The above characterizations suggested that: (1) Moss raw materials were successfully converted to the carbon material (with N and O dopants) and activated with high surface area, and (2) Sulfur particles were successfully filled into the pores of the MPC with a high loading content (58 wt %). Building on these preparations, we fabricated the Li-S battery and measured the corresponding electrochemical performances. Figure 5a shows the charge/discharge curves of the Li-S battery with the cathode material of S@MPC. The discharge processes had two plateaus near 2.28 and 2.09 V, corresponding to the reduction of sulfur and the transformation of long-chain lithium polysulfide to the short-chain lithium sulfide, respectively. The charging plateaus appeared at around 2.33 V, representing the transition from low-order lithium sulfide to higher-order polysulfide and sulfur. The initial discharge specific capacity of the S@MPC electrode was measured to be 1070 mAh g^−1^. After a functional cycle of 100, the discharge specific capacity of the S@MPC could still remain at ~708 mAh g^−1^, indicating its good cyclic stability (Figure 5b). As mentioned earlier, the discharge specific capacity of the S@MPC could be attributed to the high surface area and porosity in the MPC through NaOH treatment, implying that the dissolution of polysulfides into the electrolyte was largely alleviated by the porous structure of the MPC. Figure 5c shows the rate capability of the S@MPC electrode. At current densities of 0.1, 0.2, 0.5, 1, and 2 C, the discharge capacities of the S@MPC were found to be 832, 717, 596, 521, and 373 mAh g^−1^, respectively. The specific capacity of the S@MPC reached 763 mAh g^−1^ upon the current density return to 0.1 C. The Nyquist plots of the S@MPC electrode (Figure 5d) suggested a low charge transfer resistance, the Warburg impedance, and the ohmic resistance (including the electrolyte and electrode resistances). All these observations suggested that S@MPC with a large number of mesoporous structures could maintain reasonable specific capacity, while exhibiting great stability and rate performance.

**Figure 6:**
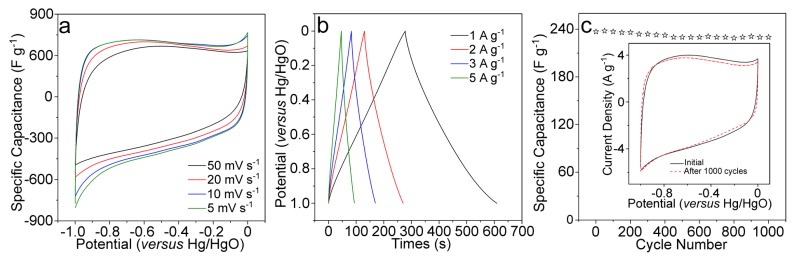
(**a**) CV curves of the as-prepared MPC electrode at different scan rates; (**b**) Charge/discharge curves of the as-prepared MPC electrode at different current density; (**c**) Cycle performance of the MPC electrode at current density of 5.0 A g^−1^.

Considering several features of the prepared MPC material, such as the porous structure with open pores, the high level of N doping, and the high specific surface area, we further evaluated its electrochemical performances as a supercapacitor. Fabrication of the supercapacitor electrode has been described in the experimental section. Figure 6a shows the CV (Cyclic Voltammetry) curves at different scan rates, which revealed the satisfactory capacitance behavior in a voltage range of −1.0 to 0 V (vs. Hg/HgO) in 6.0 M KOH electrolyte. The galvanostatic charge/discharge curve of the MPC electrode suggested a high specific capacitance of 332 F g^−1^ at the current density of 1.0 A g^−1^, which was much higher than many of other similar biomass derivated carbon electrodes (Figure 6b).

For a better comparison, some recently published data of N-doped carbon materials from biomass/biowaste are listed in Table 1. The capacitances are still high at higher current densities of 2, 3, and 5 A g^−1^, i.e., a capacitance of 237 F g^−1^ was achieved at 5 A g^−1^. We have further evaluated the cycling performance of the MPC based electrode at the current density 5 A g^−1^ (Figure 6c). During the performed 1000 cycles, the loss in specific capacitance based on the maximum value (237 F g^−1^) was less than 3%, suggesting a significantly high stability of the capacitance and the electrode. The stable electrochemical performance of the electrode was further confirmed by the similar CV curves after before and after 1000 cycles (Figure 6c, inset).

## 4. Conclusions

Moss-derived porous carbon material was evaluated as a sulfur host cathode in lithium–sulfur batteries. After being activated with NaOH, the MPC material possessed a mesoporous structure with an enhanced specific surface area and large pore volume. As a result, the S@MPC electrode gives a high discharge specific capacity, cycle stability, and rate performance. Moreover, this prepared MPC material also performed well as the electrode for the supercapacitor, giving a high capacitance and very high stability. The work here demonstrated a facile method for the utilization of moss waste biomass as alternative electrode materials for both lithium–sulfur batteries and supercapacitors.

## Figures and Tables

**Table 1:** Comparison of electrochemical performance of the biomass-derived N doped carbon materials in a three-electrode system.

Biomass Materials	Activator	C/F g^−1^	MC	Ref
Banana peel	Zinc complexes	206	1.0 A g^−1^	[36]
Shiitake mushrooms	H_3_PO_4_+KOH	306	1.0 A g^−1^	[37]
Bamboo	KOH	301	0.1 A g^−1^	[38]
Cotton	KOH	283	1.0 A g^−1^	[39]
Willow catkin	KOH	298	0.5 A g^−1^	[40]
Flour	/	261	1.0 A g^−1^	[41]
Bamboo shoot	Hydrothermal	270	5.0 A g^−1^	[42]
Agaric	KOH	324	1.0 A g^−1^	[43]
Cigarette filter	/	154	1.0 A g^−1^	[44]
Gelatin	NaOH	281	5.0 A g^−1^	[45]
Eggshell membrane	Air	297	1.0 A g^−1^	[46]
This work	NaOH	332	1.0 A g^−1^	/

C: Specific capacitance. MC: Measurement condition.
